# Testing for phylogenetic signal in claws suggests great influence of ecology on Caribbean intertidal arthropods (Acari, Oribatida)

**DOI:** 10.1038/s41598-021-83747-3

**Published:** 2021-02-23

**Authors:** Michaela Kerschbaumer, Tobias Pfingstl

**Affiliations:** Institute of Biology, University of Graz, Universitaetsplatz 2, 8010 Graz, Austria

**Keywords:** Ecology, Evolution, Genetics, Molecular biology, Zoology, Ecology

## Abstract

Claws are common biological attachment devices that can be found in a wide variety of animal groups. Their curvature and size are supposed to be parameters related to ecological aspects. Mites, known as very small arthropods, occupy a wide range of ecological niches and are a perfect model system to investigate correlations of claw morphology with ecology. There is only one study regarding this question in littoral mites but the phylogenetic impact, which plays an important role in the evolution of morphological traits, was not tested. We investigated claw shapes of different Caribbean populations of five species showing different substrate/habitat preferences. We used geometric morphometrics to quantify claw shape and tested for phylogenetic signal within this morphological trait. Even in closely related populations, we found clear claw shapes for hard versus soft substrate, confirming previous findings. Surprisingly, we found no phylogenetic signal within the trait, which demonstrates that ecology (different surfaces and substrates) has acted as one of the primary selective forces in the diversification of claw shapes. Considering that the basic claw design may be the same in the majority of arthropods, our results have important implications for further investigations of claw morphology and its ecological relevance within this phylum.

## Introduction

Claws are common biological attachment devices that can be found in a wide variety of animal groups, from tiny arthropods to birds, reptiles and even large mammals. In many of these groups they fulfill various other functions, as for example digging, climbing or grasping prey^1^. Claw features, such as curvature and size, are supposed to be parameters related to ecological aspects^2^ therefore, they were studied in several taxa. Studies on birds and reptiles, for example, demonstrated that arboreal species typically show more curved claws with a greater height than ground-dwelling species^3–5^. Based on these results, claw characteristics have also been frequently used to infer the ecology of fossil reptiles^6,7^. Most studies were exclusively performed in vertebrate taxa^8^ whereas invertebrates remained underexplored in terms of ecomorphology, though their majority possesses claws. The few existing studies demonstrated that sharper claw tips have more irregularities to interlock with and hence increase the attachment abilities of small arthropods^8–10^ but these studies did not infer a correlation with ecology. Büscher and Gorb^11^ performed a sophisticated study on the attachment devices of stick insects and demonstrated a complex complementary effect of claws and adhesive pads and that these structures correlated with substrate surface and hence with the ecology of the animals. Apart from this work, there was only a single study^12^ demonstrating a possible correlation between claws and ecology in arthropods where these authors investigated mites. Mites represent the smallest arthropods and they occupy a wide range of ecological niches, which means the landscape microstructure is highly diverse for the different ecological groups^10^. Therefore, investigating their claws may provide important insights into the correlation between morphology and ecology. Indeed, the above-mentioned study^12^ investigated the shapes of intertidal oribatid mite claws by means of geometric morphometrics and revealed a significant difference between species dwelling on hard substrates, like rock, and species dwelling on soft substrates, like mangrove roots and litter. The former show higher and stronger curved claws while the latter exhibit lower and less curved claws and species occurring on a wide range of substrates show an intermediate claw type. Intertidal oribatid mites inhabit the zone between low and high tide, which means they are subject to strong forces caused by tidal water movement. This tidal flooding causes strong selection in terms of movement and attachment resulting in the remarkable correlation between claw shape and used substrate^12^.

However, certain ecomorphological studies on other organisms^13,14^ indicated that a strong phylogenetic component can obscure correlations between morphology and ecology when comparing closely related groups. Strong phylogenetic signals have been observed in morphological structures of diverse animals, for example in the wing shape of diverse Dipterans^15,16^ or the anchor shape of parasitic Monogeneans^17^. A phylogenetic signal is defined as ‘a tendency for related species to resemble each other more than they resemble species drawn at random from the tree’^18^. In the case of intertidal oribatid mites, on the other hand, certain congeneric species clearly clustered in different ecological groups^12^ indicating that ecology is the most important factor in shaping claws and that claw shapes contain little phylogenetic information. Nevertheless, the authors of this study^12^ did not specifically test for a phylogenetic signal in their dataset, therefore, it remained uncertain if claw shape is mainly a result of ecological adaptation or just a result of closely related species showing similar ecologies.

We investigated herein the claw shapes of five different intertidal oribatid mite species from the Caribbean belonging to two different families and showing different substrate/habitat preferences. The fortuyniid *Litoribates bonairensis* and *Litoribates floridae* are distinct species which show very limited distributions and are classified as typical mangrove dwellers^19^; the selenoribatid *Thalassozetes barbara* shows a trans-Caribbean distribution and exclusively inhabits rocky habitats; and *Carinozetes bermudensis* and *Carinozetes mangrovi*, show also a wide Caribbean distribution but consist of five distinct genetic lineages, three of these lineages occur on a variety of substrates, whereas the remaining two lineages exclusively dwell either on rocky substrate or in mangrove habitats^19^.

Using this specific setup of focal species, i.e. distinct species versus closely related species/lineages with differing distributions and identical or diverging ecologies, we aimed to answer the following questions: (I) can the ecomorphological types of shape inferred by Pfingstl et al.^12^ be confirmed on different phylogenetic levels, (II) what is the exact interplay between claw morphology, microhabitat use and phylogenetic relatedness and (III) how strong is the phylogenetic signal in these claw shapes.

## Results

### Claw shape and measurements

**Figure 1:**
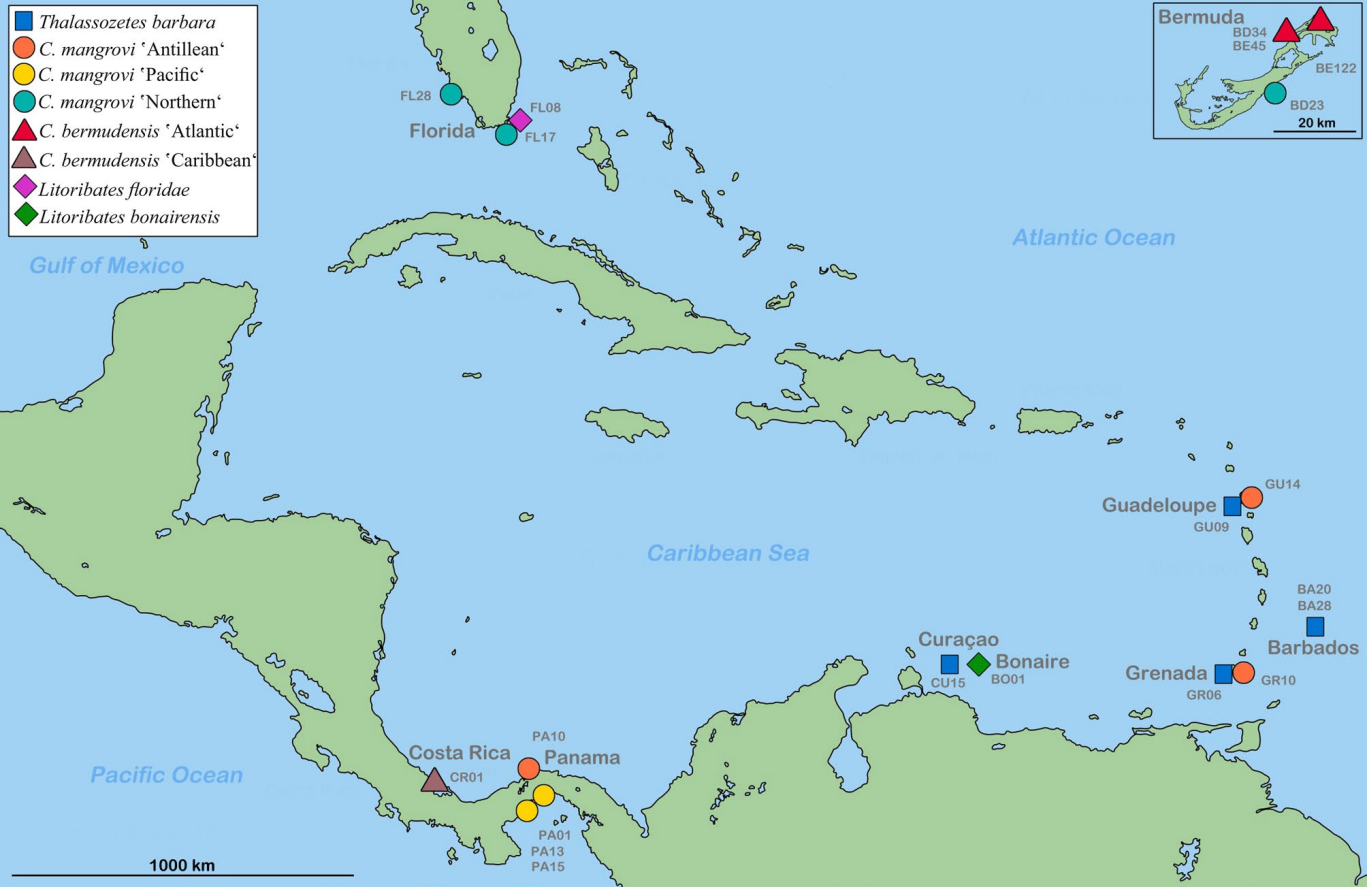
Map of the Caribbean highlighting the locations of investigated populations of Caribbean intertidal species. Genetic lineages of *C. bermudensis* and *C. mangrovi* indicated. Insert in the upper right corner showing the small island of Bermuda located in the Western Atlantic 1.600 km northeast to the coast of Florida. Codes (e.g. FL17) refer to location labels provided in Table 1. This map was created with the vector graphics editor Inkscape 0.92. (https://inkscape.org).

**Figure 2:**
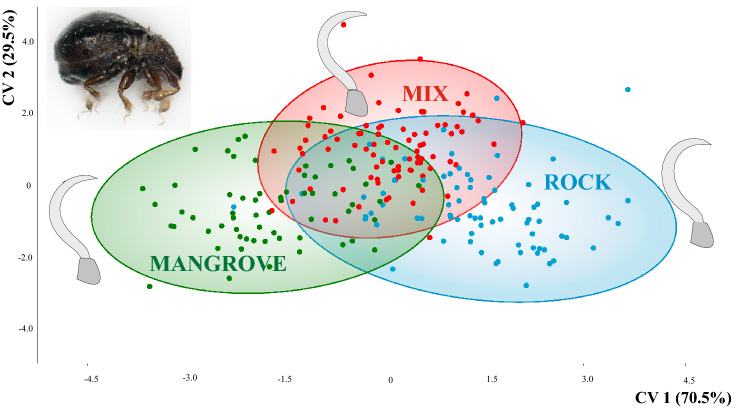
Mean claw shape in different habitats (rock, mix and mangrove). CVA scatter plot including every analyzed specimen. Lateral habitus of *C. mangrovi* shown in the upper left section of diagram.

**Figure 3:**
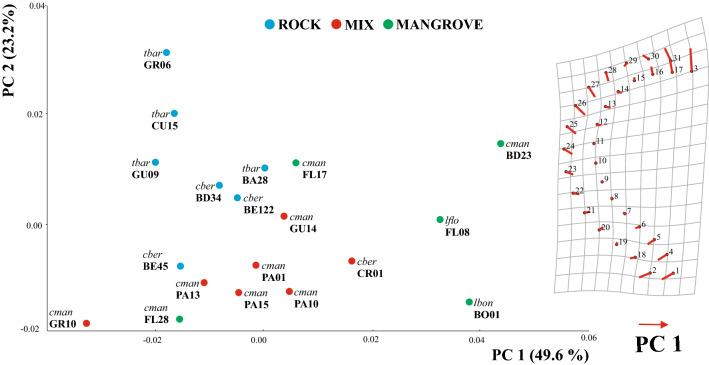
Claw shape in different habitats. PCA scatter plot of population mean shapes and deformation grid showing shape changes according to PC 1. *Thalassozetes barbara*—*tbar*, *Carinozetes bermudensis*—*cber*, *C. mangrovi*—*cman*, *Litoribates bonairensis*—*lbon*, *L. floridae*—*lflo*. Colors refer to ecotypes and codes refer to populations as given in Table 1.

**Figure 4:**
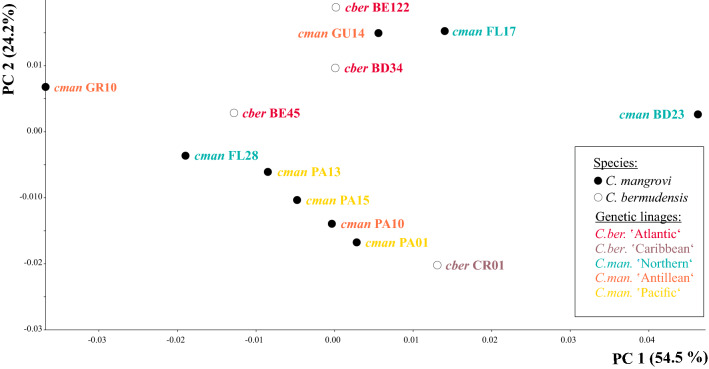
Claw shape in different species and genetic lineages within the genus *Carinozetes*. PCA scatterplot of population mean shapes of 13 *Carinozetes* populations. Colors refer to genetic lineages as shown in legend and codes refer to populations as given in Table 1.

**Figure 5:**
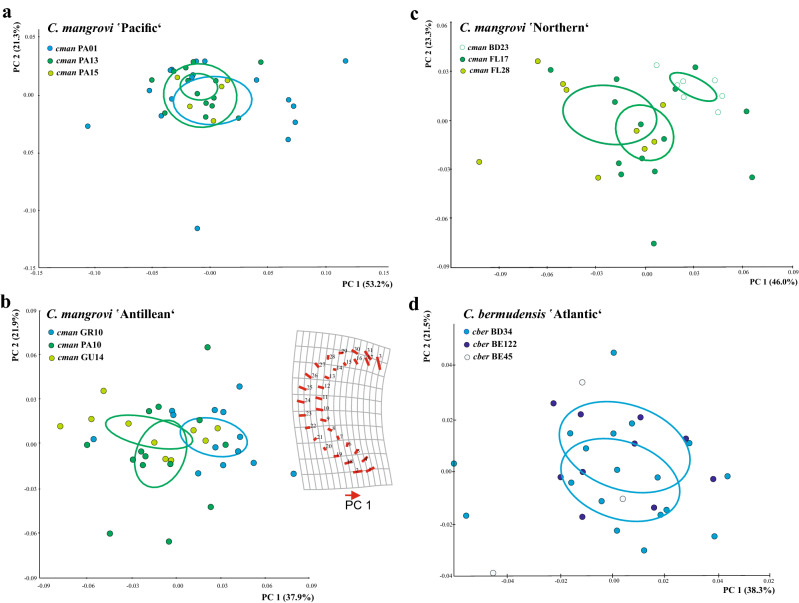
Claw shape within a genetic lineage. PCA scatter plot of specimens of (**a**) *C. mangrovi* ‘Pacific’ (**b**) *C. mangrov*i ‘Antillean’ (**c**) *C. mangrovi* ‘Northern’ and *C. bermudensis* ‘Atlantic’ with confidence ellipses for means (90% probability). Green colored points mark specimens sampled from mangroves and blue points stand for specimens originating from rocky habitats.

Our study is in line with previous findings^12^ in that CVA, including mite specimens from 19 different sampling localities (Fig. 1), based on the assignment to three habitat categories (mangrove, mix and rock) clearly revealed claw shape differentiation among diverse ecological environments. Samples from mangroves and rocky habitat separate well in the CVA scatter plot and specimens assigned to the mix type, cluster in between (Fig. 2). Depictions of the claws show mean shapes related to habitat use. Claw mean shapes (CMS) concurred well with the previously described high and strongly curved ‘rock-claw’ and the significantly lower and less curved ‘mangrove claw’. Nevertheless, shape characteristics were not that pronounced as in our previous study but given that our findings were based on more closely related populations, results made sense. Principal components 1 (PC 1) and PC 2 derived from analysis of 19 group CMS accounted for 72.8% of variation in the data (Fig. 3). CMS of the four different *T. barbara* populations clustered together at low PC 1 scores and high scores of PC 2, respectively. Claw differences among *Thalassozetes*, *Carinozetes* and *Litoribates* might be manifested in characteristics explained by PC 2. Concerning ecological impacts on claw shape, results of PCA further strengthened earlier findings that rock species show higher and stronger curved claws, especially in the distal part, while mangrove species possessed lower and less curved claws. Deformation grid in Fig. 3 showed mean shape differences in claw shape between rock and mangrove associated groups by deforming shape of average claw from negative PC axes 1 into that of positive PC axes 1. In our dataset claw shape did not reflect species classification or groupings into genetic lineages. PCA including only species from the genus *Carinozetes* showed that neither populations from the same species (*C. mangrovi* and *C. bermudensis*) nor populations belonging to the same genetic lineage clustered together in morphospace (Fig. 4). However, PCA scatter plot exposed a geographical cluster of the four populations (*cman* PA13; *cman* PA15; *cman* PA10 and *cman* PA01) from Panama. Results of PCA for claw shape in single genetic lineages are given in Fig. 5a–d. Specimens belonging to genetic lineage *C. mangrovi* ‘Pacific’ were not distinguishable in terms of sampling locality and microhabitat. Confidence ellipses for the three groups, *cman* PA01 and *cman* PA13 from rock and *cman* PA15 from mangrove roots, were completely overlapping and there was no separation among specimens from different habitats (Fig. 5a). Contrary to that plot, the PCA scatter plot of the *C. mangrovi* ‘Antillean’ lineage showed that confidence ellipses are hardly overlapping and claws of mites from mangrove habitats could be distinguished from those individuals sampled from rocks even within genetic lineage. Deformation grid within Fig. 5b illustrated that shape changes from rock type to mangrove type were changes from a more curved and broader claw to a more pointed, slender and dorsoventrally flattened claw. Our samples from the genetic lineage *C. mangrovi* ‘Northern’ were all derived from mangrove habitats but scatter plot showed separation, especially of specimens from Bermuda (*cman* BD23) and the two populations from Florida (*cman* FL17 and *cman* FL28) (Fig. 5c). All specimens from the lineage *C. bermudensis* ‘Atlantic’ were sampled from rocks and have similar claws. No separation among populations was recognizable (Fig. 5d). Traditional morphometrics, where we measured body length of every specimen in our study and related it to individual’s claw length, showed that claw length might be regulated by specimen’s body size (Figs. S1 and S2). We had variable measurements of body length, especially *T. barbara* individuals were much smaller than mites belonging to the other species. Nevertheless, the relation between body length and claw length was in the same range among all populations.

**Table 1:** Sample size (GM/MolGen), classification and information on the specific habitat for each species/lineage/population.

Origin	Code	n_Shape data_/n_genetic data_	Species/lineage	Habitat info	Ecological classification (lineage)
Barbados	BA28	8/0	*T. barbara*	*Bostrychia* on rock	Rock
Curaçao	CU15	9/0	*T. barbara*	*Bostrychia* on rock	
Grenada	GR06	13/0	*T. barbara*	*Bostrychia* on rock	
Guadeloupe	GU09	14/0	*T. barbara*	*Bostrychia* on rock	
Bermuda	BD34	19/4	*C. bermudensis* Atlantic	Diverse algae on rock	Rock
Bermuda	BE45	3/0	*C. bermudensis* Atlantic	*Bostrychia* on rock	
Bermuda	BE122	10/0	*C. bermudensis* Atlantic	*Bostrychia* on rock	
Costa Rica	CR01	14/0	*C. bermudensis* Caribbean	*Bostrychia* on rock	Mix
Bermuda	BD23	7/6	*C. mangrovi* Northern	*Bostrychia* on mangrove roots	Mangrove
Florida, USA	FL17	14/10	*C. mangrovi* Northern	*Bostrychia* on mangrove roots	
Florida, USA	FL28	9/6	*C. mangrovi* Northern	*Bostrychia* on mangrove roots	
Grenada	GR10	13/5	*C. mangrovi* Antillean	*Bostrychia* on rock	Mix
Guadeloupe	GU14	10/3	*C. mangrovi* Antillean	*Bostrychia* on dead wood	
Panama	PA10	14/6	*C. mangrovi* Antillean	Diverse algae on mangrove roots	
Panama	PA01	17/4	*C. mangrovi* Pacific	Diverse algae on rock	Mix
Panama	PA13	14/1	*C. mangrovi* Pacific	Diverse algae on dead wood	
Panama	PA15	5/2	*C. mangrovi* Pacific	Diverse algae on mangrove roots	
Bonaire	BO01	18/0	*Litoribates bonairensis*	Mangrove leaf litter	Mangrove
Florida, USA	FL08	7/0	*Litoribates floridae*	Mangrove leaf litter	Mangrove

### Phylogeny of Carinozetes

**Figure 6:**
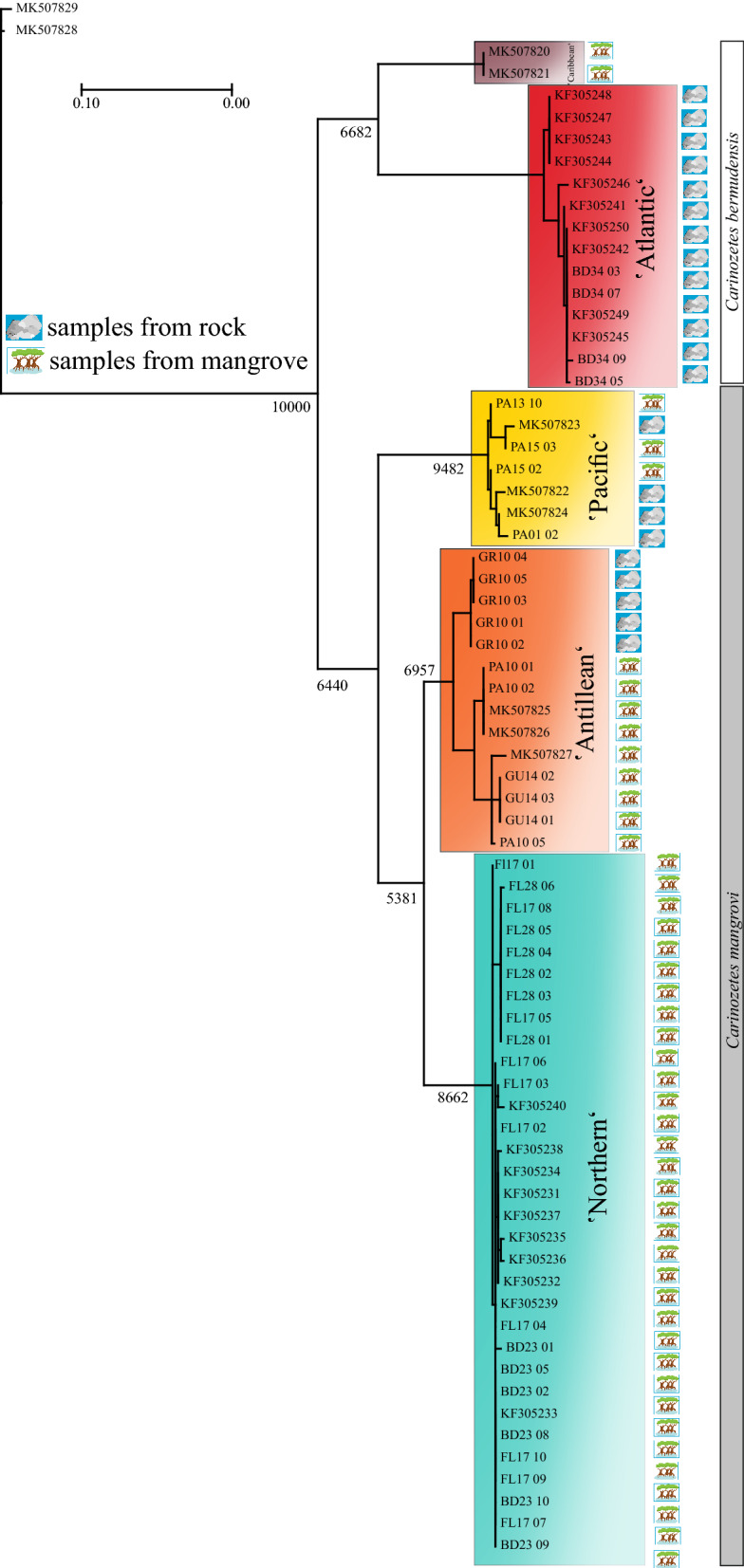
The maximum-likelihood (PhyML) phylogenetic tree inferred from COI haplotypes. Bootstrap values are indicated at major nodes (10,000 replicates). *Carinozetes trifoveatus* was used as an outgroup.

Sequences were obtained at one locus for 41 *Carinozetes* specimens. Gene bank accession numbers are given in Table S1. In congruence with the previous work of Pfingstl et al.^19^, we could confirm the existence of five genetic lineages within the genus *Carinozetes*. Maximum likelihood (ML), Bayesian inference, and neighbor-joining analyses produced largely congruent phylogenies for all clades. Phylogenetic tree derived from maximum likelihood approach with PhyML (Fig. 6) clearly demonstrate that the genus consists of two genetic lineages within *C. bermudensis*, (*Cber* ‘Atlantic’ and *Cber* ‘Caribbean’) and three lineages within the species *C. mangrovi*, namely *Cman* ‘Antillean’, *Cman* ‘Northern’ and *Cman* ‘Pacific’. With an average evolutionary divergence of 6 to 17% between species, lineages respectively and 0 to 2% within lineages our results were broadly in line with previous findings (Table 2). Interestingly despite the highest sample number, genetic variation within the Northern linage is very low with 0.45%. Phylogenetic trees constructed with Bayesian inference and neighbor-joining algorithm are given in supplementary (Figs. S3 and S4) but showed the same results as those revealed with the ML approach.

**Table 2:** Estimates of average evolutionary divergence over sequence pairs (a) within and (b) between groups (genetic lineages).

**(a)**	Antillean (14)	0.0223	**(b)**		Antillean	Atlantic	Caribbean	Northern	Outgroup
	Atlantic (14)	0.0245		Atlantic	0.1282				
	Caribbean (2)	0		Caribbean	0.1089	0.1131			
	Northern (32)	0.0045		Northern	0.0657	0.1147	0.1156		
	Outgroup (2)	0.0177		Outgroup	0.1555	0.1658	0.1615	0.1574	
	Pacific (7)	0.0108		Pacific	0.0932	0.1289	0.1154	0.1022	0.1703

### Phylogenetic signal

**Figure 7:**
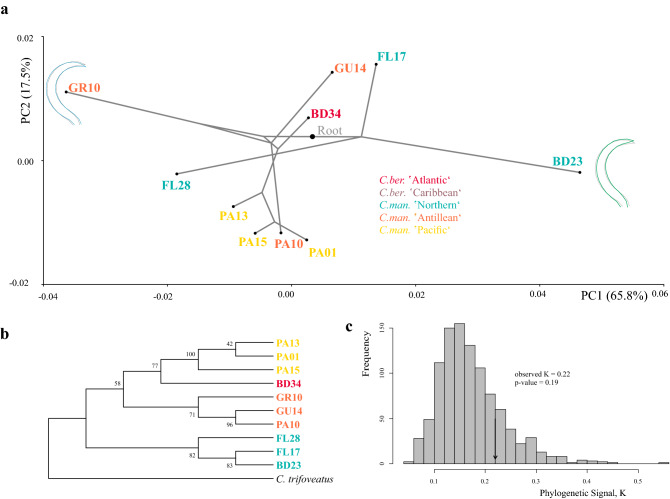
(**a**) Phylomorphospace showing changes in claw shape related to sampling location mapped onto phylogeny. Tips of the terminal branches are at the locations of population means with different colors for different genetic linages (colors are the same as in Fig. 1). (**b**) Neighbour joining tree based on COI sequences of representative individuals of 10 populations of the genus *Carinozetes* with *C. trifoveatus* as outgroup. (**c**) Histogram of *Kmult* values obtained from 999 permutations of the claw shape data on the tips of the phylogeny, with the position of observed value of *Kmult* identified.

In phylomorphospace (Fig. 7a), where the phylogenetic tree (Fig. 7b) was superimposed onto the plot of the first two principal components, populations of the same genetic lineage did not necessarily cluster together in terms of claw shape. PC 1 and PC 2 analyzed for claw shape accounted for 65.8% and 17.5% of the variation among the ten *Carinozetes* population means. Neither calculations in MorphoJ (tree length = 0.0044, P = 0.5789), nor the *Kmult* method run in R (K = 0.2205, P = 0.19) found a phylogenetic signal in claw shape for *Carinozetes* populations and genetic linages, respectively (Fig. 7c). Only the three populations PA01, PA13 and PA10 which were sampled within a small-scale geographic region around Panama, belonging to the same genetic lineage, clustered together in phylomorphospace.

## Discussion

The present results clearly confirm that there is a strong correlation between claw shape and microhabitat in intertidal mites, i.e. species dwelling exclusively on rocky substrates show considerably more curved and higher claws (higher distance between dorsal and ventral edge) than species living only on soft substrates, like mangrove roots and leaf litter^12^. All investigated taxa basically reflect this pattern, the rock inhabiting *T. barbara* shows the typical ‘rock’ claw shape, the mangrove dwelling *L. bonairensis* and *L. floridae* possess characteristic ‘mangrove’ claw shapes and the species with wider ecological ranges exhibit intermediate claw shapes. Pfingstl et al.^12^ already had included these species to their analysis so this result may not be surprising. However, they only used specimens from a single population of each species whereas the present study uses specimens from different populations, except for *L. bonairensis* and *L. floridae*, and hence demonstrates that microhabitat substrate clearly selects for specific claw shapes independent of geographic origin and local circumstances.

Morphological structures are often commensurate with phylogenetic relatedness because less morphological divergence is supposed to have occurred in closely related taxa compared with those that are more distantly related^20^. The *Carinozetes* species used in our study are closely related and were recently shown to consist of five morphologically identical but genetically distinct lineages with differing ecologies^19^, which allows us to observe the interplay of claw morphology, ecology and phylogenetic relatedness below species level.

Based on claw shapes only, neither the species nor the genetic lineages can be separated indicating a strong ecological component. Looking at the single genetic lineages, an interesting pattern is revealed suggesting that a phylogenetic relationship is though weakly represented in the claw shape. All populations of the ‘Northern’ *C. mangrovi* showed the same substrate preference, namely mangrove habitats, and their claw shapes clearly cluster according to geographic origin. In contrast, in the ‘Atlantic’ *C. bermudensis*, where all populations also occurred on a single substrate, namely rocks, no geographic pattern is reflected. Populations of the ‘Northern’ lineage were sampled from far distant locations (> 300 km apart) while populations of the ‘Atlantic’ lineage all occurred within a few kilometers (< 5 km apart). The ecologically diverse *Carinozetes* lineages show a somewhat different but though similar picture. In the ‘Antillean’ *C. mangrovi*, ecological claw types are clearly identifiable and match with the substrate used by each population. In the ‘Pacific’ lineage, on the other hand, claw shapes cannot be classified into ecological categories though each population occurred in a different microhabitat. Again, ‘Antillean’ populations originated from far distant locations (> 400 km apart) while ‘Pacific’ populations were sampled from close populations (< 50 km). Assuming that spatially close populations are closer related than more distant populations, this shape divergence between ecologically equivalent linages could reflect their phylogeny. However, mapping the shapes onto the molecular phylogeny of these *Carinozetes* species reveals a lack of a phylogenetic signal in the whole dataset as well as in each lineage. This lack is even more significant because the studied *Carinozetes* species and lineages are very closely related and in such taxa with a recent common ancestry good phylogenetic signals are likely to be found^21^. To sum these results up, analyses of molecular phylogenetic data fail to detect a phylogenetic signal in all lineages, while morphometric data fail to detect ecological groups in close geographical populations which indicates that phylogeny may still override ecology as a signal at least in very closely related populations. However, there is no doubt about the greater influence of ecology as indicated by our statistical analyses. The absence of a phylogenetic signal in a morphological structure is supposed to be mainly caused by adaption to certain factors that yield signals contrasting with molecular phylogenetic data^22^. In the present case, the loss of phylogenetic signal clearly indicates that claw shapes are mainly the result of ecological adaptation and not of phylogenetic relatedness. Why claw shapes within certain genetic lineages though correlate somehow with geography cannot be answered with the present data and needs further investigation. But one reason could be that substrate surface characteristics show greater variation across wider geographic areas, i.e., different regions may show different kinds of intertidal rocks or different mangrove species. If claws are subject to a strong selective constraint, slight changes in surface properties may already result in reducing the vital clinging abilities.

Edwards and Donoghue^23^ suggested that a lack of phylogenetic signal may be primarily an island phenomenon because islands are known for their adaptive radiations. Though many of the herein studied intertidal mites occur on separate islands, this assumption does not fully apply as one of the genetic lineages, namely the ‘Pacific’ *C. mangrovi*, is distributed along the continuous Panamanian coastline and still lacks a phylogenetic signal. Independent of geography, the intertidal habitat is an extreme environment and daily tidal flooding and wave action cause strong selection in terms of moving and attaching. Losing the grip and being washed away into the open ocean means almost certainly death for an air breathing intertidal mite and thus its claws are subject to a strong selection pressure. Morphological traits and DNA evolve at different rates, which could result in their decoupling^24^ and this may also be the case here. However, every phenotypic trait is based in the genotype and we will have to investigate the genes responsible for claw shape to understand how genotype and phenotype evolve in this specific case and environment.

To conclude, this study is the first to compare and investigate morphological traits and their phylogenetic background on family level and beyond within arthropods. Our results demonstrate that claw shape variation of intertidal mites is basically controlled by exogenous factors, i.e., microhabitat substrate properties, which drive convergent evolution. Consequently, claw shapes contain little, if any, phylogenetic information and the same may apply to other morphological structures allowing the animals to live in the intertidal environment, e.g., streamline body shape, plastron respiration etc. Our results further indicate that the evolution in extreme environments, such as the intertidal zone, the deep sea, subterranean habitats etc., may have resulted in the loss of phylogenetic signal in multiple morphological traits and taxa. Future ecomorphological studies testing for phylogenetic signals may provide us with further important clues about convergence and the evolution in highly selective environments.

## Material and methods

### Specimen source

Specimens studied in our analyses originate from the intertidal oribatid mite collection of the Institute of Biology, University of Graz (IBUG), Austria. As geographic information of studied populations is of relevance for some of our analyses, we provide some further details on the origin of populations in Table 1. Based on literature^19,25,26^ and further personal observations, species/lineages were classified into three microhabitat types (following the classification of Pfingstl et al.^12^): (1) ‘rock’—exclusively occurring in algae growing on rocky substrate, (2) ‘mangrove’—exclusively dwelling in mangrove habitats, i.e., leaf litter or algae growing on roots and (3) ‘mix’—occurring in a wider range of intertidal habitats, e.g., algae on rocks, mangrove roots, deadwood, mud etc. Classification and information on the specific habitat for each species/lineage/population is also given in Table 1.

### Geometric morphometrics

We collected geometric morphometric data from claws of 218 mite specimens, originating from 19 different localities in central America, the Caribbean islands and Bermuda (Fig. 1). The main focus of the study concentrates on populations of the genus *Carinozetes* (n = 170) and its two species *Carinozetes mangrovi (Cman)* and *Carinozetes bermudensis (Cber)*. Following the classification of Pfingstl et al.^12^, information on the specific habitat (rock, mangrove or mix), geographic information and further details on the origin of each population is given in Table 1. Because preliminary data did not show any differences in claw shape between males and females, sex was not considered in the present study. In previous work we developed a new method of assessing claw shape in mites using geometric morphometrics (GM). A detailed description of the procedure of specimen preparation, photography, the applied landmark set and the processing of coordinate data is given in Pfingstl et al.^12^. Two traditional morphometric measurements were taken from digital images, namely body length and claw length. For doing GM, claw of the first leg of each specimen was photographed and x, y coordinates of three landmarks (LM) and 32 semilandmarks were recorded by one person using TpsDig2 Version 2.31^27^.

Standard statistical analyses and analyses of shape data was done in MorphoJ^28^ and PAST PAlaeontological STatistics 3.26^29^. The geometric size of each claw was estimated by the centroid size (CS), defined as the square root of the summed squared distances of all landmarks to the centroid of the configuration^30^, transformed to log10. Landmark coordinates were aligned and superimposed by Generalized Procrustes Analysis^31^, scaling the configurations to unit centroid size and then they were centered and rotated, in order to minimize the sum of squared distances between the landmarks of each specimen to the corresponding landmarks of the mean landmark configuration of all specimens. The remaining Procrustes coordinates describe shape per se. To avoid any influence of size on shape induced through ontogenetic shape changes^32^ we performed every analysis on the residuals from a regression of Procrustes coordinates on CS^28^. To explore the degree of claw shape dissimilarity among habitats (‘mangrove’, ‘mix’ and ‘rock’) we did canonical variate analysis (CVA) on shape data including every individual claw of the dataset. CVA separates known groups in the data and provides an ordination that maximizes the separation of group means relative to the variation within groups. For each habitat we computed a mean shape wireframe graph of the claw in MorphoJ. We computed claw mean shapes (CMS) of every population in the study and performed a principal component analysis (PCA) on covariance matrix. In contrast to CVA, PCA groups are not defined a priori, allowing us to have an insight in the shape variation of claws among populations living in different habitats. To check for shape variation among the two species and the five known genetic lineages, the next analysis step was based only on populations of the same genus. PCA was done on 13 populations of the genus *Carinozetes*. For this purpose, we enlarged the existing phylogeny of the genus *Carinozetes*^26^ by genetic data of those populations we also investigated in terms of claw morphology so we could assign individuals/populations to known genetic lineages and combine morphological and genetic data (molecular genetic methods are described in the next section). Finally, we investigated claw shape within genetic entities and did a separate PCA for the lineages ‘Antillean’, ‘Atlantic’, ’Northern’ and ’Pacific’. Those analysis were based on individual claws and not on population means. For better visualization, we added confidence ellipses for means in MorphoJ. Body and claw size variability was investigated and demonstrated by box plots generated in PAST.

### Molecular phylogenetic analyses

For molecular phylogenetic analyses, total genomic DNA of 41 *Carinozetes* individuals was extracted following the rapid Chelex protocol described in Schäffer et al.^33^. Sample size and information about sampled populations are given in Table 1. We used the primers Mite COI-2F and Mite COI-2R^34^ and amplified a 564-bp fragment of the mitochondrial cytochrome c oxidase subunit 1 gene (COI) using the following PCR conditions: 3 min denaturation at 94 °C, 35–40 cycles of denaturation (30 s, 94 °C), annealing (30 s, 46 °C), extension (60 s, 72 °C) and ending with a final extension step at 72 °C for 7 min. DNA purification and sequencing steps were conducted after the methods published by Schäffer et al.^35^. Sequencing was performed in both directions on an automated capillary sequencer (ABI PRISM 3500xl, Applied Biosystems). All sequences were edited in MEGA X^36^ and aligned together with two outgroup taxa and 28 sequences of *Carinozetes mangrovi* and *Carinozetes bermudensis* available from GenBank (Table S1). The phylogenetic relationships were inferred by Bayesian inference (BI) and maximum likelihood (ML). Phylogenetic trees were constructed using MrBayes ver. 3.2.7a^37^ and a PHYML online web server^38^. Bayesian 50% majority-rule consensus tree was generated by means of MrBayes applying an MC3 simulation with 10 million generations (4 chains, 2 independent runs, 25% burn-in, GTR + I + G model). Support values for trees generated by BI were expressed as Bayesian posterior probabilities in percentages. ML analysis using PHYML was performed under the following conditions: the proportion of invariable sites was “estimated”; the number of substitution rate categories was four; the gamma distribution parameter was “estimated”; and the starting tree was a BIONJ distance-based tree. The support values of the ML tree were assessed by a bootstrap test with 1000 replicates. Tree information was visualized and edited using FigTree Version 1.3.1^39^. Results were analyzed in TRACER v.1.6^40^ to check for convergence and to ensure the stationarity of all parameters. In addition, we construct a neighbor-joining tree, which is a simplified version of the minimum evolution method and calculated the uncorrected pairwise distances with the program MEGA X within and between genetic lineages.

### Phylogenetic signal

We estimated the phylogenetic signal in our data to test if closely related individuals tend to have more similar claws to each other than of more distantly related mites. Analysis was based on a subset of specimens of four genetic lineages within the genus *Carinozetes*. We applied two different approaches. First, we followed the methods described by Klingenberg and Gidaszewski^15^. A PCA of the covariance matrix was computed from eleven population means. A reference phylogeny, a neighbor-joining tree (NJ) including one representative sequence for each population was generated in MEGA X. The phylogenetic signal contained in claw shape was evaluated by mapping this phylogenetic tree into the morphospace using squared-change parsimony. The statistical significance of the tree length value was tested with 10,000 random boot permutations of the PC scores for each population across the phylogenetic tree in MorphoJ, which simulated the null hypothesis of no phylogenetic signal in the data^15,28^. Thereby, if there is no correlation between phylogeny and morphometric data, the tree length value should be small (closer to 0 than to 1) and non-significant (*p* > 0.05).

Our second way to estimate the phylogenetic signal in claws was the generalization (*Kmult*) of the K-statistic^20^ for multivariate phenotypes^41^. Phylogenetic signal was measured by quantifying the amount of variation in the data relative to a randomized sampling of phenotypic values^42^ using R packages ‘geomorph’ version 3.3.2^43–45^ and ‘geiger’ version 2.0^46^. The significance of the K*mult* value was then evaluated through a permutation test (1000 iterations) that randomized the data across the tips of the reference phylogeny. A K-value of 1 reflects perfect accord with expected patterns of shape variance where phenotype-genotype characteristics drift due to random processes, while values greater than 1 reflect under-dispersion of variance (i.e., close relatives are more similar than expected under random processes) and values less than 1 indicate over-dispersion of variance (i.e., close relatives are less similar than expected under random processes).

## Supplementary Information

Supplementary Information.

## Data Availability

Data deposited in the Dryad Digital Repository: temporary link: https://datadryad.org/stash/share/JmQDcXJ0K8yzvRr3y_5--DQgiAbYb4a5FcA_9tZHwVM . All sequences obtained from this study were deposited in GenBank (www.ncbi.nlm.nih.gov/genbank; accession numbers in Supporting information, Table S1).
