# Supplementary material for: Testing for phylogenetic signal in claws suggests great influence of ecology on Caribbean intertidal arthropods (Acari, Oribatida)
Source: Sci Rep. 2021 Feb 23;11:4398. doi: 10.1038/s41598-021-83747-3 (PMC7902647; doi:10.1038/s41598-021-83747-3)
Supplement: Supplementary file 1 — Supplementary Information. [file 41598_2021_83747_MOESM1_ESM.pdf]

Supporting Information

Testing for phylogenetic signal in claws suggests great influence of ecology on Caribbean intertidal arthropods (Acari, Oribatida)

Michaela Kerschbaumer & Tobias Pfingstl

Table S1. List of specimens, gene bank accession number of individuals in molecular analysis

species	origin	code	specimen	ecological class.	Genbank
C. bermudensis	Bonaire	BO01	C_BO_01_2	mix	MK507821
			C_BO_01_3	mix	MK507820
	Bermuda	Be-97	Cber 97_7	rock	KF305241
		Be-97	Cber 97_15	rock	KF305242
		Be-99	Cber 99_3	rock	KF305250
		Be-120	Cber 120_2	rock	KF305243
		Be-120	Cber 120_3	rock	KF305244
		Be-120	Cber 120_5	rock	KF305245
		Be-120	Cber 120_8	rock	KF305249
		Be-120	Cber 120_9	rock	KF305246
		Be-120	Cber 120_11	rock	KF305247
		Be-120	Cber 120_14	rock	KF305248
		BD34	BD34_03	rock	MT941907
			BD34_05	rock	MT941904
			BD34_07	rock	MT941905
			BD34_09	rock	MT941906
C. mangrovi	Panama	PA01	C_PA_01_1	mix	MK507822
			C_PA_01_2	mix	MK507823
			C_PA_01_3	mix	MK507824
			PA01_02	mix	MT941908
		PA13	PA13_10	mix	MT941909
		PA15	PA15_02	mix	MT941879
			PA15_03	mix	MT941880
		PA10	C_PA_10_1	mix	MK507825
			C_PA_10_3	mix	MK507826
			C_PA_10_4	mix	MK507827
			PA10_01	mix	MT941877
			PA10_02	mix	MT941878
			PA10_05	mix	MT941876
	Grenada	GR10	GR10_01	mix	MT941881
			GR10_02	mix	MT941875
			GR10_03	mix	MT941874
			GR10_04	mix	MT941873
			GR10_05	mix	MT941872
	Guadeloupe	GU14	GU14_01	mix	MT941870
			GU14_02	mix	MT941869
			GU14_03	mix	MT941871
	Bermuda	Be-27	Cman 27_1	mangrove	KF305235

species	origin	code	specimen	ecological class.	Genbank
			Cman 27_4	mangrove	KF305236
		Be-65	Cman 65_3	mangrove	KF305231
			Cman 65_7	mangrove	KF305232
			Cman 65_8	mangrove	KF305234
		Be-97	Cman 97_1	mangrove	KF305237
			Cman 97_2	mangrove	KF305238
			Cman 97_8	mangrove	KF305239
		Be-125	Cman 125_3	mangrove	KF305240
		Be-128	Cman 128_1	mangrove	KF305233
		BD23	BD23_01	mangrove	MT941903
			BD23_02	mangrove	MT941901
			BD23_05	mangrove	MT941900
			BD23_08	mangrove	MT941899
			BD23_09	mangrove	MT941902
			BD23_10	mangrove	MT941898
	Florida	FL17	FL17 01	mangrove	MT941897
			FL17 02	mangrove	MT941896
			FL17 03	mangrove	MT941895
			FL17 04	mangrove	MT941894
			FL17 05	mangrove	MT941893
			FL17 06	mangrove	MT941892
			FL17 07	mangrove	MT941891
			FL17 08	mangrove	MT941890
			FL17 09	mangrove	MT941889
			FL17 10	mangrove	MT941888
		FL28	FL28 01	mangrove	MT941887
			FL28 02	mangrove	MT941886
			FL28 03	mangrove	MT941885
			FL28 04	mangrove	MT941884
			FL28 05	mangrove	MT941883
			FL28 06	mangrove	MT941882
C.trifoveatus			BD 10 2	OUTGROUP	MK507828
			BD 10 3	OUTGROUP	MK507829

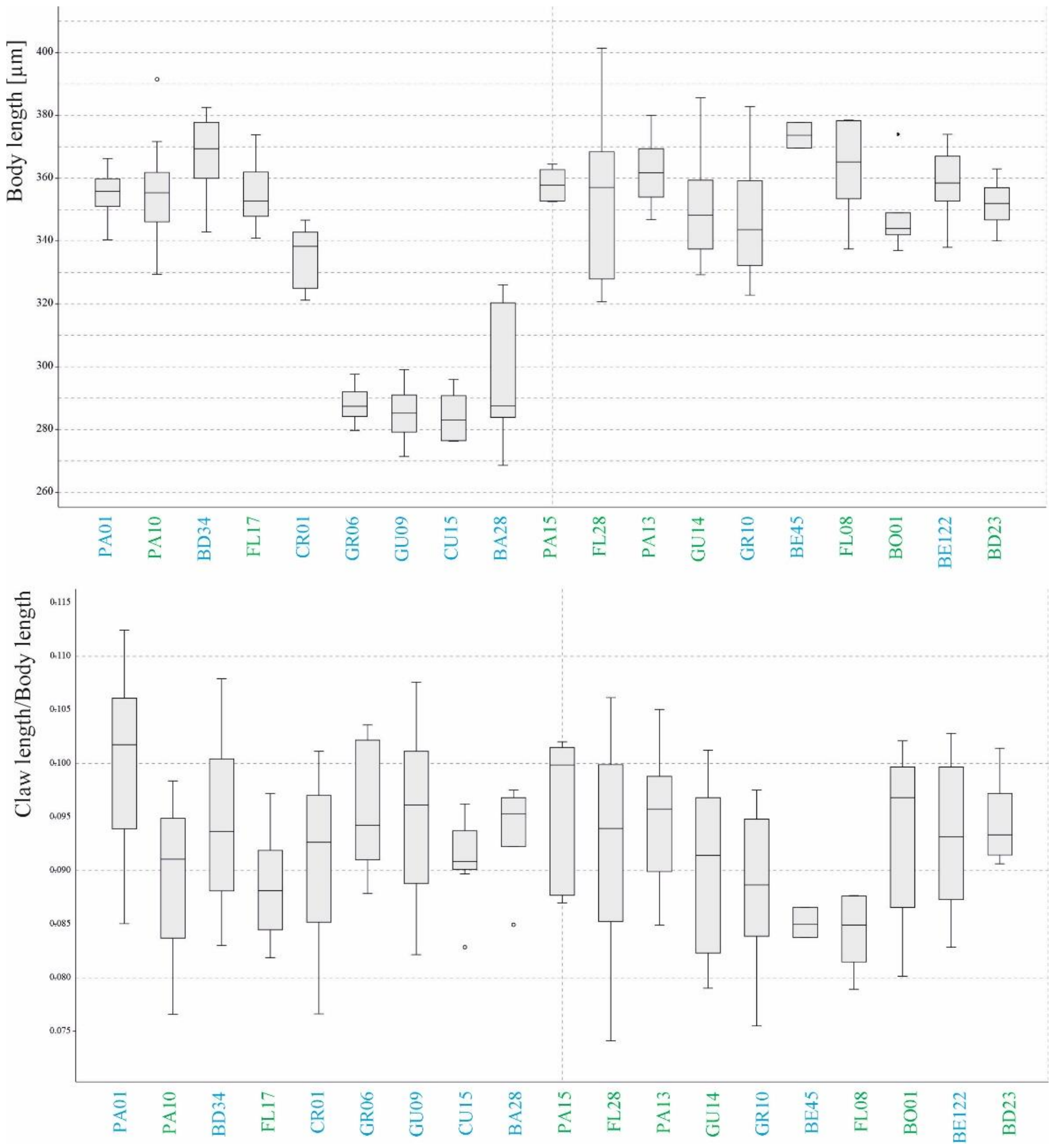

Figure S1. Box plots showing body and claw size variability between investigated populations. The box plots present medians, 25 and 75 percentiles; limits are the 95% confidence intervals. Outliers are represented as dots. Green colored population names are populations sampled from mangroves and blue stand for specimens sampled from rocky habitats.

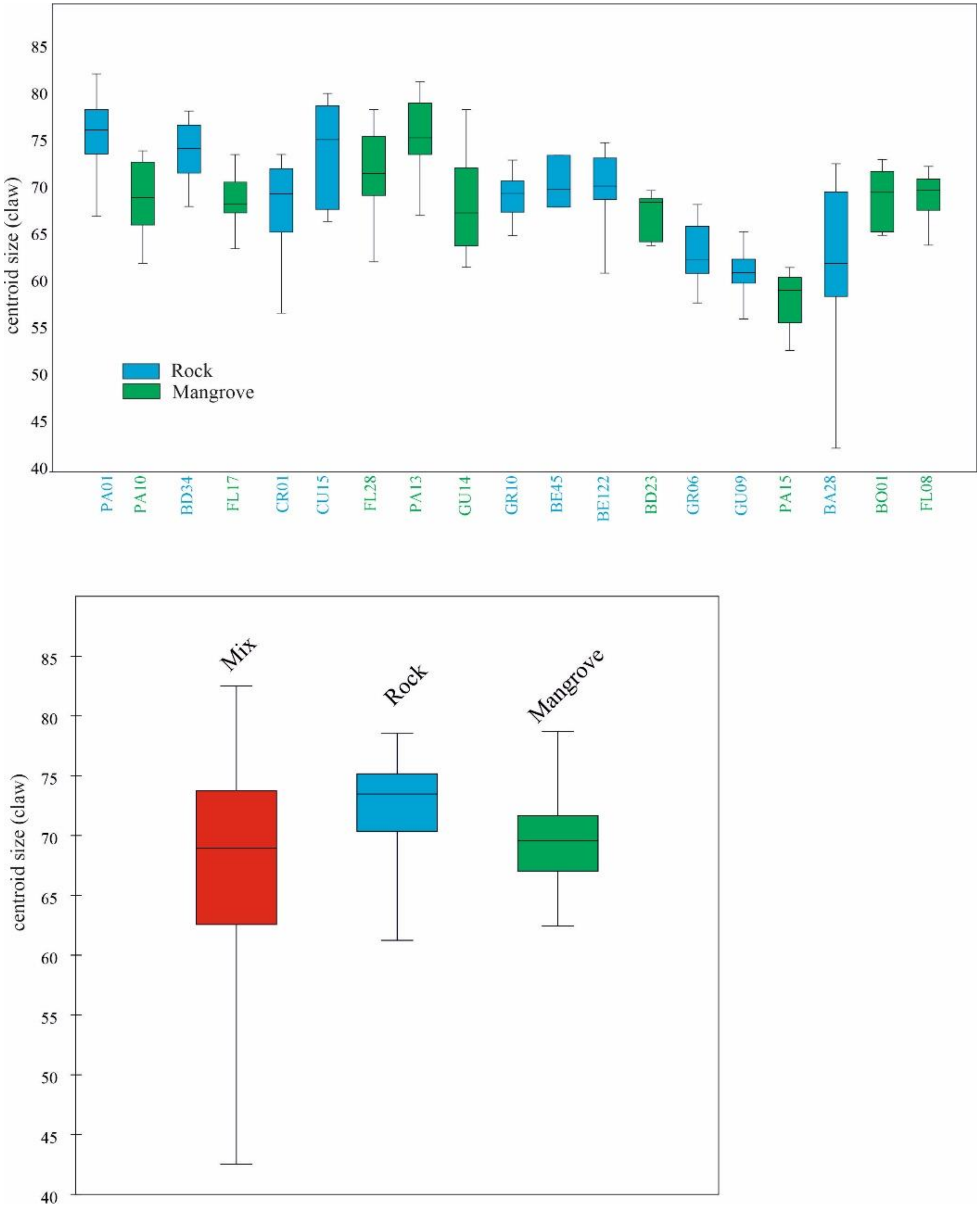

Figure S2. Box plots showing variability in centroid size between investigated populations and habitats. The box plots present medians, 25 and 75 percentiles; limits are the 95% confidence intervals. Outliers are represented as dots. Green colored population names are populations sampled from mangroves and blue stand for specimens sampled from rocky habitats.

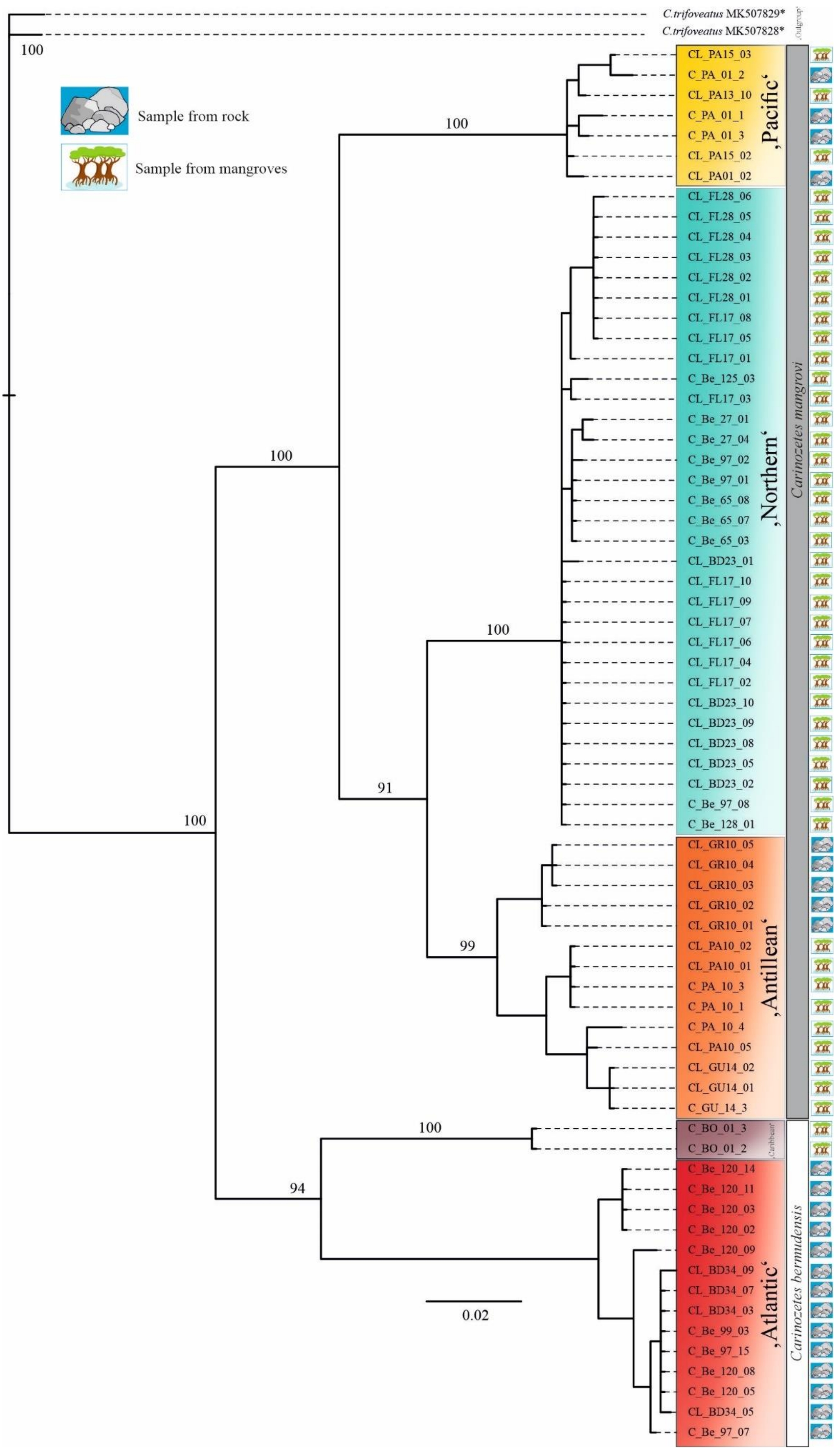

Figure S3. Phylogenetic tree constructed with Bayesian inference (BI) analysis. Bootstrap values are indicated at major nodes.

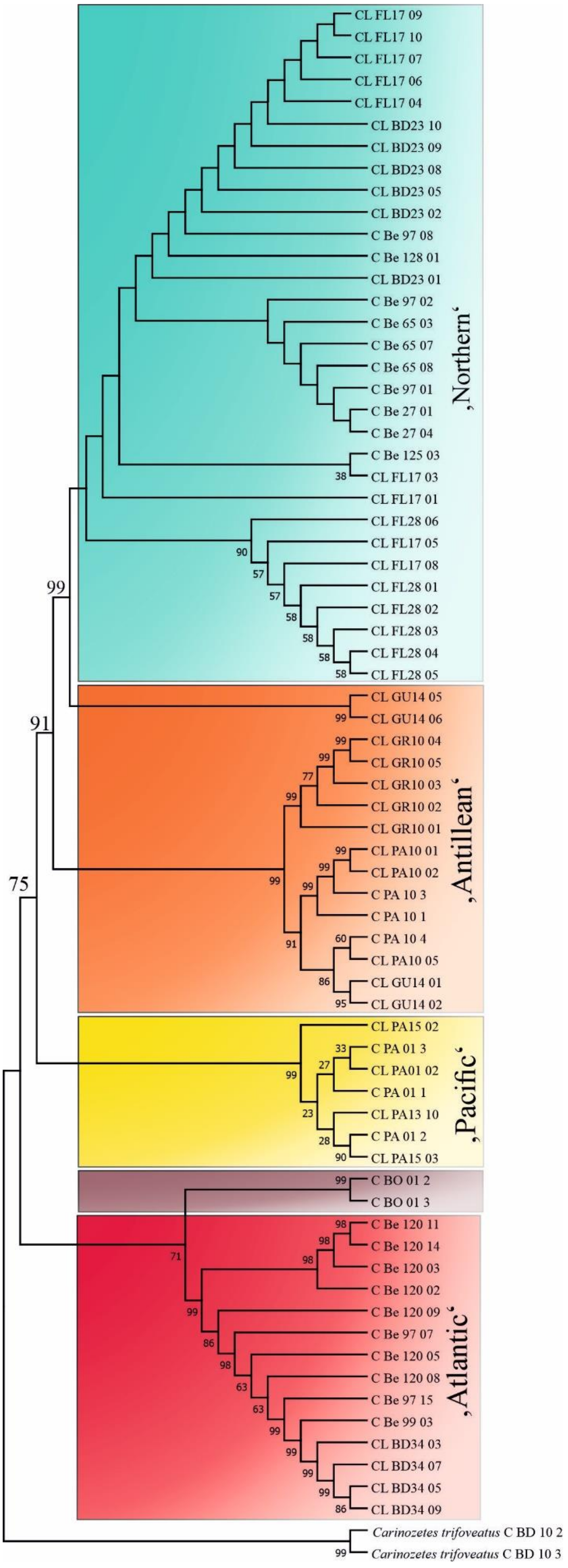

Figure S4. Phylogenetic tree constructed with Neighbor joining algorithm. Bootstrap values are indicated at major nodes.
